# Regulating Biocompatibility of Carbon Spheres via Defined Nanoscale Chemistry and a Careful Selection of Surface Functionalities

**DOI:** 10.1038/srep14986

**Published:** 2015-10-14

**Authors:** Santosh K. Misra, Huei-Huei Chang, Prabuddha Mukherjee, Saumya Tiwari, Ayako Ohoka, Dipanjan Pan

**Affiliations:** 1 Beckman Institute for Advanced Science and Technology, University of Illinois at Urbana-Champaign, Department of Bioengineering and Materials Science and Engineering, Urbana, 61801, USA; 2 Department of Chemistry, University of Illinois at Urbana-Champaign, Urbana, 61801, USA; 3 Carle Foundation Hospital, Urbana, 61801, USA

## Abstract

A plethora of nanoarchitectures have been evaluated preclincially for applications in early detection and treatment of diseases at molecular and cellular levels resulted in limited success of their clinical translation. It is important to identify the factors that directly or indirectly affect their use in human. We bring a fundamental understanding of how to adjust the biocompatibility of carbon based spherical nanoparticles (CNPs) through defined chemistry and a vigilant choice of surface functionalities. CNPs of various size are designed by tweaking size (2–250 nm), surface chemistries (positive, or negatively charged), molecular chemistries (linear, dendritic, hyperbranched) and the molecular weight of the coating agents (MW 400–20 kDa). A combination of *in vitro* assays as tools were performed to determine the critical parameters that may trigger toxicity. Results indicated that hydrodynamic sizes are potentially not a risk factor for triggering cellular and systemic toxicity, whereas the presence of a highly positive surface charge and increasing molecular weight enhance the chance of inducing complement activation. Bare and carboxyl-terminated CNPs did present some toxicity at the cellular level which, however, is not comparable to those caused by positively charged CNPs. Similarly, negatively charged CNPs with hydroxyl and carboxylic functionalities did not cause any hemolysis.

A myriad of innovations in nanotechnology has made a range of novel therapeutic and imaging probes possible. Nanoparticles (NPs) have been shown to target cells and tissues as imaging agents and to release drug by subsequently dropping overall dosages as well as controllable, acceptable toxicity^1^. Accordingly, wide ranges of core and surfactants compositions with tunable sizes and shapes for these nanomaterials are utilized predominantly in biomedical imaging^2^, diagnostics^3^, treatment^4^ and the control of biological systems^5^. Despite of those promising characteristics, the translation of nanomaterials into clinical usage has unfortunately been impeded by their contentious, contradictory results of toxicity^6^,^7^. The unique characteristics of NPs present challenges of additional safety issues arising from the interactions of cellular and other blood borne constituents during systemic circulation and reticuloendothelial system (RES) clearance. The intrinsic properties of NPs are vastly different from their small molecular counterpart, and, therefore, are also subject to the scrutiny of the immune system. In particular, the complement system is a rapid-acting, first-line host defense mechanism that defends the intravascular space and other biological compartments from foreign invaders and cellular debris^8^. Complement recognizes potential targets, marks them for clearance and/or lysis, and initiates inflammatory reactions. For example, a clinically approved liposome-encapsulated doxorubicin suspension (Doxil®) is known to activate the complement system with serious clinical implications^9^. It is, therefore, of immense importance to fundamentally understand what might trigger systemic as well as cellular toxicity and how defined chemistry is linked to the size, shape, surface chemistry, capping agents, and degree of aggregation of the nanomaterials.

The progress in chemistry, molecular biology, engineering and medicine has shaped the biomedical research to underline the importance of early detection and treatment of diseases at the molecular and cellular level. Myriads of nanometer-sized architectures have been proposed, developed and tested in laboratory and in preclinical models. However, very few have been translated for human use. It is, therefore, a major task to identify the factors that directly or indirectly affect their eventual application in human. The current understanding of biological and biophysical obstacles encountered by the nanometric agents are manifold, including but not limited to external barrier (skin), en route barriers (serum stability, opsonization, immune response, systemic toxicity etc.) and cellular barriers (endosomal entrapment, cellular toxicity etc.). A detailed understanding of these parameters and how defining surface chemistry may influence their biological interaction is vital and may promote the development of next generation nanotechnologies for translational and clinical applications. Towards this aim, the goal of this manuscript is to use combination of *in vitro* assays as tools to outline the destiny and response of CNPs with respect to adoptive surface functionalities. Understanding of their structure-toxicity relationship will help to design particles to reduce their systemic and cellular toxicity. Carbon nanoparticles are designed by tweaking their size (2–250 nm), surface chemistries (positive, or negatively charged), varying molecular chemistries (linear, branched and hyperbranched polymers) and the molecular weight of the coating materials (MW 400 Da–20 kDa). These parameters were used to modulate the surface of CNPs without changing the core of the particles. In a nutshell, we proposed a methodology to produce carbon particles and studied how their systemic and cellular level toxicity can be manipulated by surface chemistries. To the best of our knowledge, this approach is entirely novel and has not been explore in the past.

Of late, carbon nanospheres are gaining widespread attention as they enable both *in vitro* and *in vivo* imaging and therapeutics by serving as an optical probe that allows absorption and emission of photons at a specified wavelength. It has also emerged as a vehicle that encapsulates, adsorbs or attached a therapeutic agent to significantly increase the payload for controlled drug delivery^2^. However, a fundamental understanding of their biocompatibility is vastly unexplored. To elucidate the biocompatibility of nanoplatforms, effects of their chemico-physical characteristics on immune system in addition to their cellular response are important indicators of their overall safety. Despite the fact that numerous studies have shown a strong correlation between complement activation and the size^10^, morphology^11^, and surface configuration^11^ and modification of functional groups of nanocarriers^12^, the understanding of diverse1nanomaterials that trigger strong or negligible complement is mostly confined to liposomes^13^, polymeric^14^ and metallic NPs^14^. To date, few have shown the complement recognition and activation induced by carbon-based nanoplatforms-carbon nanotubes and carbon black only^15^. We, herein, report cellular and systemic toxicity results of unrevealed CNPs. We explore the effects of diverse sizes, capping agents, surface available functional moieties and electrophoretic mobility on cellular toxicity and innate immunocompatibility.

## Results and Discussion

### Preparation and characterizations of CNPs

**Figure 1:**
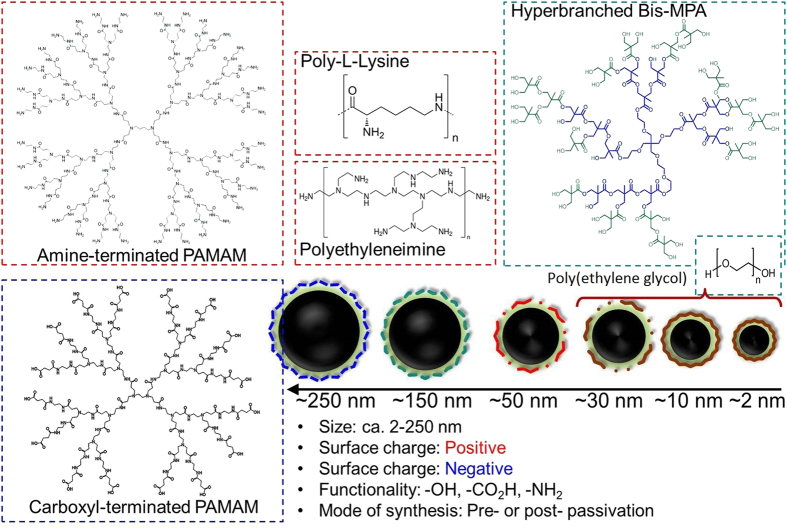
Surface charge, functional and morphological parameters. Linear, branched (dendritic) and hyper-branched macromolecules were used as pre- and post- passivated agents, resulting either in positive or negative surface functionalities. Sizes of CNPs synthesized in this work vary from approx. 2 to ≈250 nm.

Two distinct synthetic methodologies were adopted. For both the syntheses of pre-and post-passivated nanomaterials, a natural carbohydrate, e.g. nectar agave, was used as an inexpensive carbon source whose surface was passivated with linear and branched organic macromolecules. Agave nectar, also called as agave syrup, is a natural sweetener commercially produced from several species of agave, including Agave tequilana (blue agave) and *Agave salmiana*^16^. Agave syrup is typically sweeter than honey and less viscous. Linear poly(ethylene glycol) (PEG) (M_*n*_ = 400, 1000, 4,600, 10,000 and 20,000 Da) and poly-L-lysine (PolyLys) were applied for an *in situ* passivation of pre-and post-synthesized nucleating agents, respectively. Branched macromolecules utilized in this study include (1) hyperbranched bis-MPA polyester hydroxyl polymers (Bis-MPA), (2) amino-and (3) carboxyl-terminated PAMAM dendrimers (PAMAM+ and PAMAM−, respectively) and (4) branched polyethyleneimine (PEI25K) as shown in Fig. 1. To compare the influence of surface charges over cellular and systemic toxicity, neutral, negative and positive charged macromolecules were chosen: PEG 400–20k, and Bis-MPA are neutral, PAMAM− are negatively charged, whereas PolyLys, PAMAM+ and PEI25K are positive macromolecules. PEG 400–20k is a linear polyether compound with one hydroxyl group at each end. Bis-MPA is pseudo fourth generation macromolecule (G4) contains 64 peripheral hydroxyl groups. PAMAM− , a succinamic acid dendrimer, is a 1,2-ethylenediamine core (G3.5) equipped with 64 number of carboxylic acid groups. In contrast to negatively charged PAMAM, PAMAM+ also equipped with a 1,2- ethylenediamine core (G5) corresponding to 128 number of amino groups was used as a comparison of opposite surface charges. Similarly, poly-L-lysine, another amino acid polymer having approximately one HBr per lysine residue was investigated as well. Finally, branched PEI was used as a repeating unit composed of primary, secondary, tertiary amine groups with two carbon aliphatic CH_2_CH_2_ spacers. The synthesis of the pristine (bare, uncapped) and pre-passivated CNPs is cost-effective and involved only a simple hydrothermal step using a commercial hot plate (detailed procedures can be found in Methods). In a typical synthesis, commercial grade nectar agave (batch composition; 47–56% of fructose and 16–20% of glucose by weight, the rest is other sugars and water) was suspended with the passivating agent (≈10 wt%), purged with argon and heated for 10–20 min. A 1:10 ratio (w/w) of agave-to-polymer was found to be the ideal composition. Other ratios (e.g. 1:2, 1:4, 1:6, and 1:8) were also explored but resulted in poor dispersibility presumably due to the insufficient *in situ* capping of the nucleating carbon core by polymers. The use of a higher carbohydrate to polymer ratio 1:20 was also investigated, but it did not result in any noticeable improvement in dispersion or overall colloidal stabilities. Prolonged reaction time (>30 min) formed insoluble charred material seemingly due to the volatility of the organic macromolecules under that drastic condition.

### Sizes and electrostatic distribution

**Figure 2:**
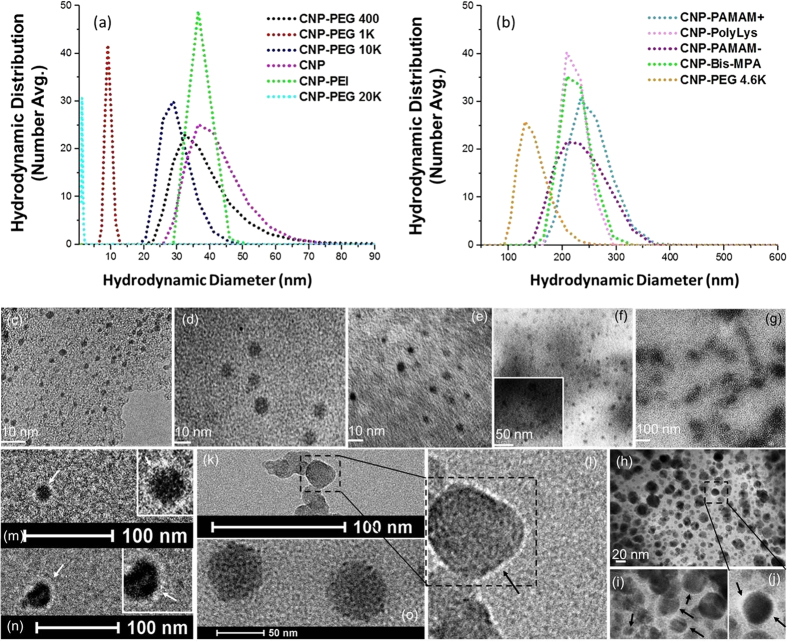
Hydrodynamic and anhydrous diameters of CNPs. (**a**) Hydrodynamic diameters increase from 1 to ≈46 nm in the order of CNP-PEG 20 K, CNP-PEG 1 K, CNP-PEG 10 K, CNP-PEG 400, CNP-PEI and CNP. (**b**) Hydrodynamic sizes from DLS increase from ≈139 to ≈250 nm in the order of CNP-PEG 4.6 K, CNP-Bis-MPA, CNP-PAMAM−, CNP-PolyLys, and CNP-PAMAM+. (**c**–**h**) Transmission electron microscopy (TEM) images of CNPs: (**c**–**e**) pre-passivated CNP-PEG 400, CNP-PEG 4.6 K and CNP-PEG 10 K, respectively. (**f**–**h**) post-functionalized CNP-Bis-MPA, CNP-PEI and CNP-PolyLys, respectively. Representative TEM images to depict the presence of outer passivating polymer layers from CNP-PolyLys (**i**,**j**), CNP-PEG 10 K (**k**,**l**), CNP-Bis-MPA (**m**), CNP-PEG 4.6 K (**n**) and CNP-Pristine (**o**).

Based on our chosen functional and morphological parameters, we observed these particles ranging from ca. 2 to 250 nm. To the best of our knowledge a systemic approach to produce CNPs within that size range is not known. The hydrodynamic diameters of CNPs passivated with PAMAM+, PAMAM−, PolyLys and bis-MPA were found to be significantly larger than the other CNPs (Table 1 and Fig. 2a,b) attributed to the larger bushy chains, which are extended once these particles are suspended in aqueous media. On the other hand, a more tightly wrapped thin layer of the polymer was envisioned for CNP-PEGs and CNP-PEI. These particles were further analysed by transmission electron microscopy (TEM) in an anhydrous state shown in Fig. 2c–o.

TEM analyses for carbon particles are always challenging due to their lack of inherent contrast. For TEM analyses, 10 *μ*L aliquot of the diluted CNP stock solution was placed on 400 mesh copper grid. After one minute, excess fluid was removed by a piece of filter paper. DLS measurement, shown in Fig. 2a,b and in a tabular form in Table 1, revealed that the CNPs are monodispersed and the hydrodynamic size in aqueous suspension can be tailored to particles within 2–250 nm. Significant variation in hydrodynamic sizes was noticed as a function of passivating agents and synthetic protocols (pre- and post-passivation). The typical anhydrous morphologies of CNPs are exhibited in Fig. 2c–o. TEM image showed that the as synthesized CNP-pristine (no passivating agent/surface coating) appeared as spherical particles with good monodispersity. The core sizes of the CNPs were estimated to be in good agreement with their hydrodynamic distribution sizes. As they are capped within the various passivating polymers, each CNP revealed a bright corona around the particle with a thickness ranging from ca 1.5–4.5 nm. Figure 2i–n show the representative TEM images of CNPs (CNP-PEG, CNP-PolyLys, CNP-Bis-MPA) as uniform spherical nanoparticles with distinct presence of surface coating.

Studies of electrophoretic mobility indicate that CNPs capped with PEGs, Bis-MPA and PAMAM− have negative zeta potential (Table 1). In contrast, particles enclosed by PAMAM+, PolyLys, and PEI resulted in positive values of zeta potential (26 ± 5, 16 ± 6 and 9 ± 1 mV, respectively).

### Optical properties and aqueous dispersibility

**Figure 3:**
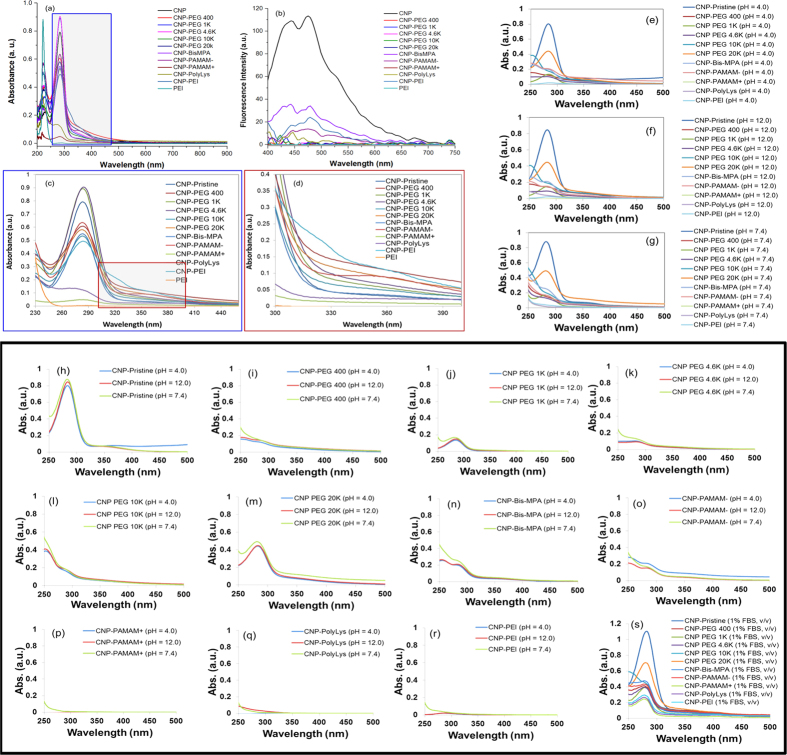
UV-Visible and fluorescence spectra of CNPs. (**a**) Absorbance of UV-Vis spectra were collected at an interval of 1 nm from 200 to 900 nm. (**b**) Emission spectra of fluorescence covered an excitation range between 400 and 750 nm. (**c**,**d**) Expanded area of interest from UV-Vis absorption spectra. UV-vis spectroscopic details of CNPs compared at various H + maintained at pH 4.0, 7.4 and 12. All the CNPs were found to have decreasing absorption efficiencies in pH 4.0 and 12.0 compared to absorption at pH of 7.4. UV-vis spectroscopic details of CNPs compared at pH of 4.0, 7.4 and 12. All the CNPs were found to have decreasing absorption efficiency in pH 4.0 and 12.0 compared to absorption at pH of 7.4. Specifically, CNPs with positive surface functionalities, i.e. CNP-PAMAM+ (**p**), CNP-PolyLys (**q**) and CNP-PEI (**r**) decreased the CNPs absorption to maximum extent.

The ultraviolet-visible (UV-Vis) absorption spectra of coated CNPs and that of CNP-Pristine show a weak, broad intensity around 350 nm accompanied by a strong absorption at ≈300 nm which represents the distinct signature of CNPs (Fig. 3a). Branched PEI, by contrast, is in the absence of this signature.While, it is evident that all the carbon nanoparticles strongly absorb in ca. 270 nm range, surface passivation with macromolecules clearly alters their optical properties causing a bathochromic shift in their absorption spectra. Further closer look revealed that molecular weight and surface charge might be influencing the change of spectral band position in the absorption spectrum to a longer wavelength and lower frequency. With increase in molecular weight of the passivating polymer, a strong absorption ca. 360 nm was noticed, while cationic polymers influenced a red shifted spectral band still maintaining the signature absorption band at 270 nm. This can presumably happen due to the change in environmental conditions and as a result of change in polarity at a molecular level. A further systematic investigation is highly warranted to fully understand the plausible effect of surface passivation over the absorptive behavior of carbon nanoparticle. This work is beyond the scope of this study which more geared towards biocompatibility and structure toxicity relationship.

To understand the fluorescence properties, CNPs were excited at *λ*_*ex*_ 365 nm and their emission was monitored at *λ*_*ex*_ 400–750 nm. Noticeably, as shown in Fig. 3b negatively charged CNPs passivated with branched macromolecules Bis-MPA and PAMAP- result in significantly higher fluorescence than their negatively charged, linear PEG-passivated counterparts. We believe that this observation is owing to different surface chemistry. As a passivating agent that induces negatively surface charge becomes more extensively branched (linear to G4), the average radiant efficiency increases considerably because of an increase in surface area available for light passivation. In addition, negatively charged, branched CNPs possess higher radiant efficiencies than linear PEG equivalents that contains fewer electron donating groups, showing that enriched optical properties are likely due to the greater number of electron donating groups from dendritic or hyperbranched coating as well. Interestingly, there exists an opposite effect upon the relationship between the fluorescence properties and the increasing surface area when taking account of positively charged CNPs. Unlike more light emitted from more branched, negatively charged CNPs, greater diminution of fluorescence is found in positively charged CNPs with increasing surface area and the number of electron withdrawing groups. The choice of surface chemistry between linearity and branches combined with the amount of electron donating or withdrawing groups makes circumventing auto-fluorescence from biological samples and achieving a deeper penetration toward the target possible.

The CNPs synthesized in this work are desired for systemic biomedical application. In order for them to be suitable for use, it is critical for them to have well dispersibility/suspendibility in aqueous medium. We performed UV-vis spectroscopic experiments to correlate suspendability of CNPs with their surface functionalities. All the CNPs were suspended in aqueous medium of pH 4.0, 7.4 and 12.0 at same concentration before acquiring the absorbance by UV-vis spectroscopic measurements (Fig. 3e–g). Comparing all the CNPs at pH 4.0, 7.4 and 12 individually, turned out to be minimally effective in reducing the absorption efficiency of all the CNPs (Fig. 3h–o). But CNPs with positive functionalities (Fig. 3p–r) caused maximum decrease at pH 4.0 and 12.0 compared to pH of 7.4 probably due to significant decrease in suspendability of the CNPs at pH 4.0 and 12. It revealed the reduction in dispersibility of CNPs at pH 4.0 and 12.0. Addition of 1% FBS (v/v) in all the CNP suspensions found to be improving suspendability of all the CNPs with increased absorption efficiency (Fig. 3s)

### Molecular fingerprints and stability

**Figure 4:**
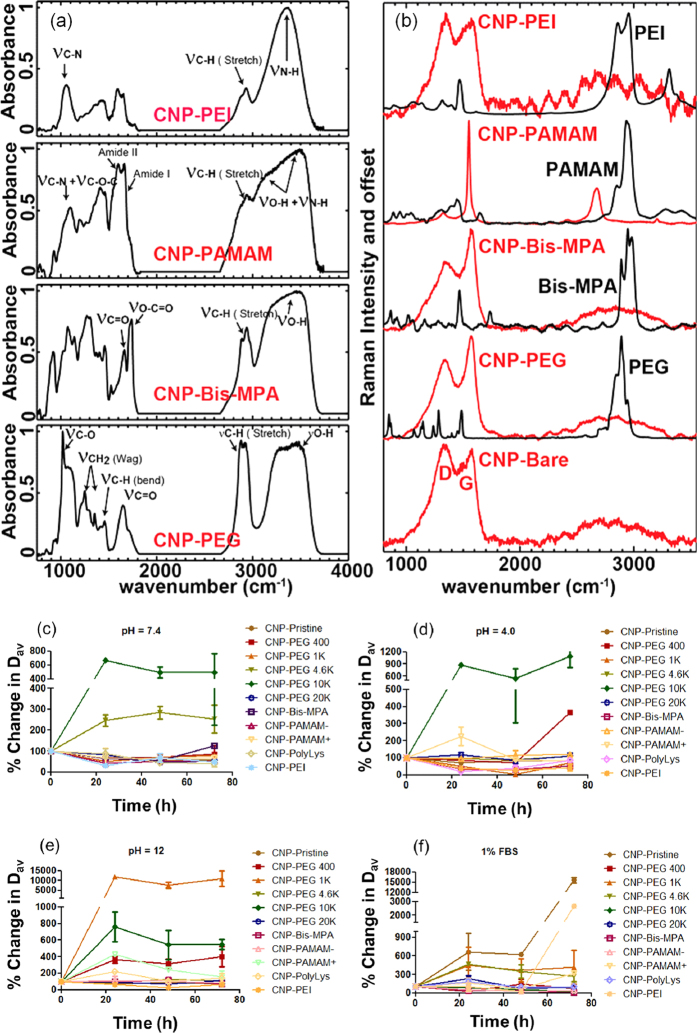
Chemical bonding and molecular structures identified by FT-IR and Raman spectroscopy. (**a**) FT-IR traces of CNP-PEI, CNP-PAMAM−, CNP-Bis-MPA, CNP-PEG, respectively (top to down). (**b**) Overlaid Raman spectra of PEI and CNP-PEI, PAMAM− and CNP-PAMAM−, Bis-MPA and CNP-Bis-MPA, PEG and CNP-PEG and CNP-bare, respectively (top to down). (**c**–**f**) Stability of CNPs in acidic, neutral and basic pH and in presence of fetal bovine serum (FBS). Particle stability was followed by hydrodynamic diameter determination at various time points including 0, 24, 48 and 72 h. Dav = hydrodynamic diameter.

Chemical bonding and molecular structures of CNPs were assessed by FT-IR and Raman spectroscopy. Figure 4a shows the infrared spectra of CNPs passivated with various ligands PEG, Bis-MPA, PAMAM− and PEI. Each of the passivated CNPs exhibits infrared spectra corresponding to the ligands as well as their carbon core. It has been suggested earlier that the surface of carbon nanomaterials have dangling hydroxyl or carbonyl moieties that can be identified via infrared spectroscopy. We observe carbonyl stretching (–C=O), C–O, C–H (stretch and bend) and broad O-H vibrations for CNP-PEG and C–N, C–H (stretch and bend) and N–H vibrations for CNP-PEI. In addition, Acetyl (O–C=O), carbonyl, and broad O-H vibrations, and amide I and II modes are present in CNP-Bis-MPA and CNP-PAMAM−, respectively. Figure 4b shows the Raman spectra of CNPs. Shown in red are the Raman spectra of the bare and passivated CNPs. Each of the Raman spectra is characterized by the “G” (graphitic) and the “D” (disordered graphitic) modes as expected in amorphous carbon materials. Although none of the Raman spectra shows the characteristic features of the ligands (shown in black), we note that the intensity ratio of the G and the D bands (I_*G*_/I_*D*_) progressively increases from CNP-Pristine < CNP-PEG < CNP-Bis-MPA CNP-PAMAM− and then falls down to the same value as CNP-PEI. The incident laser radiation is near resonant to the *π*-π* transition of the graphitic modes, which generates a strong polarization field due to the strong electron-phonon interaction. This polarization overwhelms any polarization generated due to vibrational Raman scattering (weak) of the ligands and thus does not exhibit in the spectra. However, the impact of the surface chemistry due to passivation is reflected in the increasing intensity ratio (I_*G*_/I_*D*_) of the aforesaid materials that suggests a steady rise in the graphitic domains in them. Spherical carbon nanoparticles are known to have oxidized carbohydrate functionalities on particle surface including hydroxyl, aldehydes, carboxylic acid and carboxylate ions^17^. We investigated the presence of these functionalities by correlating surface charge potential by zeta potential measurement (Table 1). For post passivated CNPs, hydrogen bonding and electrostatic interactions are presumably playing a major role between CNP surface functionalities and passivating molecules used for coatings. For pre-passivated CNP preparation, similar interactions can be envisioned in addition to some un-identified forces that played role to induce seeding of nanoparticles. Further mechanistic studies are warranted, however that is beyond the scope of this work. Stability of functionalized CNPs was investigated at varying pH 4.0, 7.4 and 12.0. Particle integrity was also studied in presence of 1% serum to pre-evaluate the acute fate of CNPs in systemic circulation. Experiment was performed at 37 °C over a period of three days. Particle stability was followed by the change in hydrodynamic diameter distribution at four time points including 0, 24, 48 and 72 h. All the CNPs were used in same concentration for all the pH variants and serum stabilities. Prerequisite for developing stable colloidal suspension for every biomedical application is an appropriate surface functionalization of such nanoparticles, which will dictate their interaction with the environment. These interactions eventually affect the long term colloidal stability of the particles. All the CNPs were found to be stable along the time points of investigation with no considerable changes noted in hydrodynamic diameter except for CNP-PEG 10 K and CNP-PEG 4.6 K at physiological pH of 7.4 (Fig. 4c). In case of CNP-PEG 10 K and CNP-PEG 4.6 K, presumably the particles aggregated to produce bigger assemblies of 600 nm and 250% larger size. A change in pH to acidic 4.0, led to only significant change in particle size of CNP-PEG 10 K while CNP-Bis-MPA found to be aggregated only at 72 h time point (Fig. 4d). Rest of the particles were found to be stable across the incubation period. Further change in H+ ion concentration with pH to 12, destabilized CNP-PEG 1 K along with CNP-PEG 10 K while CNP-Bis-MPA (Fig. 4e). On the other hand, presence of 1% fetal bovine serum caused significant aggregation in positively charged surfaces of CNP-PEI particles probably due to strong electrostatic interactions with negatively charged serum proteins. As expected, CNP-Pristine was found to be aggregated probably due to absence of any surface coating which resulted in lack of inter-particle repulsive forces and hydrogen bonded interlinking between particles (Fig. 4f). The results support the basic understanding of colloidal stability of nanoparticles. The functionalities present on the nanoparticle surface not only dictate the nucleation of the particles during synthesis, but also prevent the aggregation in suspension. The repulsive forces between particles in suspension, in principle, produced due to the presence of a hydration layer on the surface, electrostatic repulsion or even steric exclusion. Depending on the passivated system, the choice of appropriate ligand may help to stabilize particles. The passivating molecules are first bound to the carbon surface by some attractive interaction, either chemisorption, electrostatic attraction or non-covalent hydrophobic interaction, uniquely presented by the functional moieties present. Studying *In vivo* stability of these particles was not under the scope of current study but on course for future studies. Our preliminary experiments on similar particles were found to be significantly compliant for *in vivo* applications^18^.

### Safety profile of CNPs, dependence on surface functionalities and cellular response

**Figure 5:**
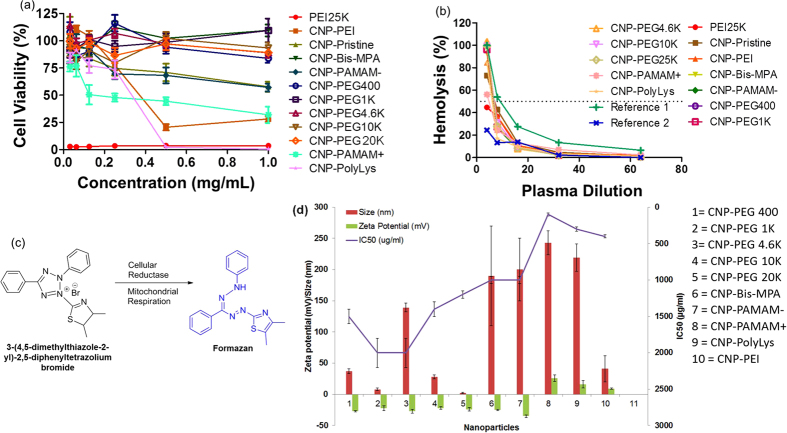
Cellular and systemic toxicity evaluated by MTT and CH 50 assays. (**a**) The cytotoxic effects on C32 human melanoma cells after incubation of PEI and CNPs for 48 hr. (**b**) Immunotoxic assessment of complement activation resulted from 4, 8, 16, 32 and 64 times dilutions of swine plasma. (**c**) Change in chemistry of 3-(4,5-dimethylthiazole-2-yl)-2,5-diphenyltetrazolium bromide after cellular reduction in living cells. Formazan concentration correlates with viable cell population among treated pool of cells. (**d**) Comparative evaluation of IC50 values related to hydrodynamic diameters and surface zeta potential of various nanoparticles.

Molecular and cellular imaging is recognized as noninvasive techniques to see cellular and sub cellular events at a very early time point. The potential role of nanoparticles in imaging and drug delivery is unquestionable. For the past two decades, this area has gained remarkable attention with high potential for clinical translation. The potential of nanoparticles for both detection and drug delivery has been well documented. Major advancement has been made towards the development of defined nanoparticles for conducting dual function i.e. imaging and therapy (theranostics). However, their clinical translation is still elusive and a significant effort need to be devoted to gain better understanding of their interaction with biological systems. Carbon based nanoparticles are gaining boost for various biomedical applications due to their unique optical properties. However, to fully exploit their potential, a thorough understanding of their biocompatibility is highly desired. An investigation on their safety profile and composition toxicity relationship (CTR) would provide valuable information to help in their translation. Cellular toxicity was investigated by evaluating decrease in mitochondrial respiration correlating with decrease in % alive cells. Mitochondrial respiration eventually leads to mitochondrial oxidation involves in redox process with added MTT (3-(4,5-dimethylthiazole-2-yl)-2,5-diphenyltetrazolium bromide) leads to reduction of MTT and producing blue colored formazan crystals (Fig. 5c). Concentration of produced formazan crystals found to be directly proportional to the live cell population which decreases with increased cellular toxicity of treated formulation or CNPs. An UV-vis spectroscopic measurement of solubilized formazan crystals compares the % viable cells after a particular CNP treatment revealing the cellular safety of particle.

In current study, chemical combination of CNPs with surface modulating molecules has been investigated for toxicity studies. In terms of individual toxicity effects of these molecules in nanoparticle forms have been studied well similar to studies on CNPs^19^. Role of molecular chemistries from PEI, PEG400–20 K, Bis-MPA, PAMAM−, PAMAM+ and PolyLys has been studied independently for cellular toxicity studies. PEIs generally exhibit significant *in vitro* and *in vivo* toxicity as free, uncomplexed PEI molecules interact with negatively charged serum proteins and even red blood cell surfaces^20^,^21^. Its toxicity also dependents on molecular-weights and branched nature of PEIs where higher molecular weight and branched PEIs of same molecular weight showed higher cellular toxicity^22^,^23^. Similarly, molecular weights of PEI decide the complement activation during systemic circulations or interaction with blood serum proteins^24^. Generally, pegylation result in stealth shielding and increased circulation times of nanoparticles in various bioactive delivery protocols probably due to the formation of a dense hydrophilic barrier of PEG chains on the surface of the carrier reducing the interactions with the reticular-endothelial system (RES)^25^. Although PEGs are generally safe for cellular toxicity, some forms of PEG-phospholipids might cause activation of the complement system and potentially cause pseudo-allergic reactions^26^. Dendrimers are known to be activating complement pathways by exposure to blood serum^27^ while extent of complement activation depended on the hierarchy of poly(amidoamine) (PAMAM)^28^. On the other side, cytotoxic action of PAMAM dendrimer was correlated with the number of primary amino groups^29^, and decreased with reducing number of primary amines^30^ while type of surface charge also decided the toxicity and complement activation^31^. Poly 2,2-bis (methylol) propionic acid (Bis-MPA) materials have been reported as non-toxic, non-immunogenic, and biodegradable materials *in vivo* drug delivery systems^32^. These macromolecules in their nanoforms may exhibit cellular and immunotoxicity based on molecular weight, functional groups and their respective charges. It was presumed that cellular and immunotoxicity of CNPs should also be influenced with such variables when these macromolecules are used as passivating agents.

Cell viability in the presence of PEI and CNPs was evaluated by a MTT assay upon C32 human melanoma cells. PEI25K alone shows significantly higher toxicity with an IC_50_ less than 30 *μ*g/mL in comparison to CNP-PEI with an IC_50_ of 400 ± 20 *μ*g/mL, which has been found to be most responsive to cell viability compared to the other CNPs (Fig. 5a and Table 2). CNP-PolyLys and CNP-PAMAM+ also cause significant decreases in viability (IC_50_ = 300 ± 30 and 100 ± 20 *μ*g/mL, respectively). Albeit CNP-bare and CNP-PAMAM− induce some toxicity, the overall percentages of viability are much higher than those of the positive charged CNPs and no corresponding IC_50_ can be obtained. PEG-and Bis-MPA-coated CNPs yield remarkable inertness in C32 cells with an IC_50_ more than 1000 *μ*g/mL.

### Immunocompatibility

Compared to cellular toxicity, immune system cellular responses endanger the biocompatibility of nanoplatforms profoundly. Immunity is stratified into two domains: On one hand, innate immunity is an immediate defense, however, lack of specificity against foreign molecules; on the other hand, adaptive immunity is a precise control of antigen-specific reactions, but requires up to several weeks to initiate^33^. Complement, one of innate immune systems, composed of more than 30 proteins either on cell surfaces or in plasma is activated by three pathways—classical, mannan binding lectin, and alternative pathways^34^. Strikingly, all three pathways involve a cascade of amplification steps with a convergence C3^35^. Accordingly, the highlight of complement in immunity is non-negligent. One foreign molecule induces the activation of a complement protein (e.g., C3) that is cleaved into several fragments (C3a and C3b), leading to a significant multiplication of the next activation products and introducing opsonization, osmotic lyses and clearance of the foreign target, and ultimately stimulation of inflammation responses^36^,^37^,^38^.

A CH50 assay is a screening test for an activated classical pathway and is found to be sensitive to the reduction, absence and/or inactivity of any component based on the lysis of sensibilized sheep erythrocytes in the presence of Ca^2+^ and Mg^2+^. CH50 values are the serum dilution factor that represents 50% lysis of antibody-sensitized sheep red blood cells. Among various drug vehicles, PEI25K is known for triggering immune defense mechanisms *in vitro* and *in vivo* as well as PEI polyplexes through binding to plasma proteins and activating the complement^39^,^40^,^41^. We explored the systemic toxicity related to PEI and CNPs with plasma samples collected from swine blood (refer to Methods for a detailed procedure of plasma collection). Two references were used: Reference 1 was plasma with normal complement and reference 2 was, in contrast, with low complement. We found that except positively charged PEI, CNP-PEI, CNP-PAMAM+ and CNP-PolyLys, all the other formulations were unable to activate complement proteins. There were no significant changes in the values of CH50 for CNP-PEGs, CNP-Bis-MPA, and CNP-PAMAM− with respect to normal compelment level plasma (8 ± 1 for all negatively charged CNPs vs. 9 ± 1 obtained from reference 1/control 1 as shown in Table 2). In contrast, PEI, CNP-PEI, CNP-PAMAM+ and CNP-PolyLys resulted in CH50 values of 2 ± 1, 5 ± 1, 5 ± 1 and 6 ± 1, respectively that lean towards lowering the level of the complement close to reference 2 (CH50 = 2 ± 1). Our finding that PEI has the strongest reactivity of inducing immune response is consistent with the complement activation-related pseudo allergy found by Merkel *et al.*^40^. Among all the positively charged CNPs, CNP-PolyLys shows the least reactivity, however, found to have higher reactivity than counter charged CNPs.

The primary purpose of the manuscript was to use combination *in vitro* assays as tools to outline the fate of CNPs with respect to various surface functionalities. A better understanding of their structure-toxicity relationship will help to design particles minimizing their systemic and cellular toxicity. Furthermore, we throw some light onto how these particles respond to en route barriers such as blood serum. Various molecular chemistries were used to modulate the surface of CNPs without changing the core of the particles. In short, the overarching goal of this work was to suggest a methodology to produce carbon particles and to define how surface chemistries may affect their systemic and cellular level toxicity. A straightforward chemical pathway is introduced to develop CNPs with various functionalities and particle sizes ranging from 2–200 nm. At a certain concentration of macromolecule mixed with CNP source, particles of particular size were produced with known surface chemistry which were finally used for verifying various physico-chemical and spectroscopic properties. It ended with investigating cellular toxicity and eventually complement activation respect to surface functionalities.

In short, we bring a fundamental understanding of the biocompatibility of CNPs through defined chemistry and a careful selection of surface functional groups. There have been studies that show complement activation as well as cellular toxicity induced by carbon nanotubes; no extensive studies, nevertheless, have been reported on the effects of size, charge, morphology and functional properties of spherical CNPs on blood complement and cell viability. In spite of no systemic synthetic approach that was used to derive spherical CNPs with tunable sizes, our pre- and post- surface passivated methods have brought in diverse sizes ranging from the lowest 2 up to 250 nm. In addition, the variation of surface charge and morphology determined by the amount of electron donating or withdrawing groups and the choice between linearity and branches enable the modification of their optical properties. Followed by physicochemical characterizations, we looked into the critical parameters that generate detrimental effects at the cellular and systemic level. Results indicate that sizes are not a risk factor for cellular and systemic toxicity, whereas the presence of a highly positive surface charge generates significant complement and cellular toxicity (Fig. 5d and Table 2). CNP-bare and carboxyl-terminated CNPs did present some detrimental effect at the cellular level which, however, is not comparable to those caused by positively charged CNPs. Similarly, negatively charged CNPs with hydroxyl and carboxylic functionalities did not cause any hemolysis. Our studies reveal that a cautious fabrication of nanoplatforms regulates their biological interaction, which may open a panel of designing more effective imaging modalities and controlled drug delivery.

## Methods

### Materials

Polyethylenimine, branched (PEI, average M_*w*_ ≈ 25,000), hyperbranched bis-MPA polyester-64-hydroxyl (Bis-MPA, generation 4, ≥97%, M_*w*_ = 7,323.32), PAMAM dendrimer, ethylenediamine core (PAMAM−,generation 3.5 solution, sodium carboxylate surface groups, 10 wt. % in methanol, M_*w*_ = 12927.69), PAMAM dendrimer, ethylenediamine core (PAMAM+, generation 5 solution, amino surface groups, 5 wt. % in methanol, M_*w*_ = 28824.81), poly-L-lysine (PolyLys, M_*w*_ ≈ 150,000–300,000, 0.1% (w/v) in H_2_O), poly(ethylene glycol) (PEG, average M_*n*_ = 400, M_*n*_ = 950–1,050, M_*n*_ = 4,600, M_*n*_ = 10,000 and M_*n*_ = 20,000) and sucrose (≥99.5%) were purchased from Sigma-Aldrich (MO, USA) and used without further purification. Agave nectar (18% glucose and 56% fructose by weight, HoneyTree’s® Organic Agave Nectar, MI, USA) was obtained from a local grocery store.

### Preparation of CNPs

Pristine CNPs were prepared by dissolving 1 g of agave nectar in 1 ml of nanopure water (0.2 *μ*M, 18 MΩ·cm). Then the aqueous solution was heated on a hot plate at 300 °C for roughly 1 hr and resuspended in 3 ml of nanopure water. Followed by 2-minute probe sonication (Q700^*TM*^, Qsonica Sonicators, CT, USA) (Amp:1, On: 2 sec, Off: 1 sec), the solution was passed through a syringe filter with a 0.2 *μ*m pore size (Millex®, Merck Millipore Ltd., County Cork, Ireland) prior to use. In order to make CNP-PEI, 10 *μ*L of branched PEI solution were added to 1 mg of CNPs. After vortexed and left at room temperature for 1 hr, the solution was centrifuged for 30 min at 75,000 rpm at 4 °C (Optima^*TM*^ MAX-XP Ultracentrifuge, Beckman-Coulter, CA, USA) to remove the suspension. The collected pellet was then redispersed in nanopure water and probe sonicated for 20 sec (Amp: 1, On: 2 sec, Off: 1 sec). CNP-Bis-MPA, CNP-PAMAM−, CNP-PAMM+ and CNP-PolyLys were synthesized by mixing a 1:10 ratio (w/w) of CNPs to hyperbranched bis-MPA polyester-64-hydroxyl, carboxyl- and amino-terminated PAMAM dendrimers, and poly-L-lysine, respectively. CNP-PEG 400, CNP-PEG 1 K, CNP-PEG 4.6 K, CNP-PEG 10 K and CNP-PEG 20 K were prepared via the following method. A mixture of agave nectar and polymer (or Agave nectar alone) was taken in glass sample vial of 20 mL capacity. To this, was added nanopure water and using a long glass rod was mixed well to form a homogenous admixture with or without the presence of passivating agents. The concentration of agave nectar to polymeric passivating agent was maintained at 1:10 (w/w). A 1:10 ratio (w/w) of nectar agave-to-polymer was found to be the optimal to produce adequate colloidal stability. Other ratios (e.g. 1:1, 1:2, 1:4) were also explored but resulted in poor solubility; which can be attributed to insufficient coverage of the carbon core by the passivating polymer. For the nucleation process, a hot plate surface was maintained at 270 °C while glass vials were kept un-capped to allow slow evaporation of water. The temperature was maintained for 10–15 min which led to generation of brownish to black mass (varies with passivating agent). The reaction mixture visually changed color from light yellow to dark brown to black. The as synthesized particles were centrifuged at 12000 g for 20 min, followed by collecting the supernatant by filtering through a 0.22 *μ*m syringe filter.

### Size and Zeta potential Measurements

The hydrodynamic diameters and surface charges of CNPs were determined by a Malvern Zetasizer Nano ZS90 (Malvern Instruments Ltd., Worcestershire, UK). The averaged hydrodynamic diameter was obtained from the peak values of the number distribution with 3 ≤ n ≤ 6 except only two measures available for CNP-PEG 20k and results were reported as mean ± standard deviation. Transmission electron microscopy (TEM) imaging was collected by JEOL 2010 LaB6 and JEOL 2100 Cryo (JEOL Ltd., Tokyo, Japan). Samples were deposited on 200-mesh Quantifoil®holey carbon grids (Structure Probe, Inc., PA, USA) to visualize their anhydrous morphology.

### UV-Vis spectroscopy and Fluorospectrometry

Ultraviolet-visible (UV-Vis) absorbance of CNPs was recorded via GENESYSTM 10S UV-Vis Spectrophotometer (Thermo Scientific, MA, USA). Absorbance spectra were collected at an interval of 1 nm from 200–900 nm. The emission spectra of fluorescence measurements were obtained through NanoDrop 3300 Fluorospectrometer (Thermo Scientific, MA, USA). The excitation maximum was set to 365 nm and the wavelength covered an excitation range between 400 and 750 nm.

### Raman and FT-IR spectroscopy

Aqueous solution of the particles was dried onto MirrIR IR-reflective glass slides (Kevley Technologies, Chesterland, Ohio, USA) for Fourier Transform Infrared (FT-IR) measurements using a PerkinElmer Spotlight 400 (PerkinElmer, Waltham, Massachusetts, USA). For each measurement 100 × 100 *μ*m images were collected at 8 cm^−1^ spectral resolution with 8 scans per pixel and a 25 × 25 *μ*m pixel size and individual spectra were corrected for atmospheric contributions. Raman spectra were acquired through dried samples described in the Infrared spectroscopy section in a reflection mode of Horiba LabRAM 3D (Horiba, Japan). The excitation wavelength for all measurements was 532 nm and the power was set to 25 mW with a 10 s acquisition time. The Raman shift from 400 to 4000 cm^−1^ was collected at 8 cm^−1^ spectral resolution. Laser light was focused through a 100×, NA 0.95 objective into the sample plane and the scattering was collected in the reflection geometry using the spectrograph coupled with an Andor Newton back-illuminated EMCCD camera.

### MTT assay

The cytotoxic effect of PEI and those of CNPs were investigated with a 3-(4,5-dimethylthiazole-2-yl)-2,5-diphenyltetrazolium bromide (MTT) assay (Sigma-Aldrich, MO, USA). C32 human melanoma cells were plated on 96-well plates at a density of 8,000 cells per well (Greiner Cellstar® 96 well plates, Sigma-Aldrich, MO, USA) and exposed to PEI and CNPs after 24 hr at 37 °C. Followed by a 42 hr exposure time, 20 *μ*L (5 mg/mL) of MTT solution was added to each well and cells were further incubated for another 4.5 hr. After exposure, media were aspirated and 200 *μ*L of dimethyl sulfoxide (DMSO, ≥99%, MP Biomedicals, USA) was added to dissolve formazan crystals. Optical density of the samples was determined by Synergy HT (BioTek, USA) with a reference wavelength of 630 nm. The percentage cell viability was determined by the following equation:


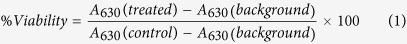


### CH50 assay

The systemic toxicity was evaluated by HaemoScan Complement CH50 Assay Kit (HaemoScan bv, Groningen, Netherlands) using the manufacturer’s protocol. In brief, 100 *μ*L of erythrocyte suspension was added slowly to 0.5 mL of Dilution Buffer and the solution was gently tumbled and centrifuged without a cap on for 10 min at 400 × g (Eppendorf Centrifuge 5810R, Eppendorf, NY, USA). The cell pellet was collected by aspirating its supernatant and multiple centrifugations may be required. The pellet was resuspended in 4 mL of Dilution Buffer. Plasma samples were collected from swine blood according to the following general plasma collection protocol. Plasma was exposed to various formulations at 10% (v/v) for 10 min at 37 °C, and then was centrifuged at 2000 rcf for 10 min. The supernatant was collected, serially diluted to 4, 8, 16, 32 and 64 times and 50 *μ*L of each diluted supernatant was withdrawn to a new centrifuged tube. Dilution Buffer was used as a negative control while lysis fluid was a positive control. Two references were used: Reference 1 was plasma with normal complement and reference 2 was, in contrast, with low complement. References were also used in dilutions range from 4 to 64 times.

Reference dilutions and controls (50 *μ*L each) were added to centrifuged tubes followed by the addition of 50 *μ*L of the erythrocyte suspension to each sample. After Samples were incubated for 30 min at 37 °C, 100 *μ*L of stop solution (Life Technologies, NY, USA) was added to all tubes. All samples were centrifuged at 400 × g for 10 min. Supernatants (100 *μ*L) were collected and transferred to 96-well Microtiter^*TM*^ Microplates (Thermo Scientific, MA, USA) and the optical density of the samples was determined by Synergy HT with a reference wavelength of 415 nm. CH50 values were obtained by plotting the % Lysis values against dilution factor while % Lysis was calculated by the formula:


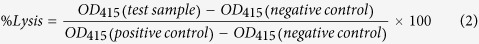


Statistics were done by One-Way ANOVA with Dunnett’s post test, keeping reference 1 as a control. The significance level was presented by P values. A value of P < 0.05 was considered statistically significant.

## Additional Information

**How to cite this article**: Misra, S. K. *et al.* Regulating Biocompatibility of Carbon Spheres via Defined Nanoscale Chemistry and a Careful Selection of Surface Functionalities. *Sci. Rep.* **5**, 14986; doi: 10.1038/srep14986 (2015).

## Figures and Tables

**Table 1:** Physico-chemical characteristics of as-synthesized and post-capped CNPs in hydrated states.

Nanoparticles	Descrition	Precursor: passivator ratio (w/w)	Dispersibility	Preparation temperature, time*	Post synthesis processing**	DLS (nm)	Zeta (mV)	PDI	Functionality	Surface charge
**CNP-Pristine**	No-passivation	1:0	Water	270 °C; 30 min	Probe sonication; filtration	46 ± 17	−17 ± 02	0.26	Bare, Carboxylate, –OH, etc.	Negative
**CNP-PEG 400**	Pre-passivation	1:10	water	270 °C; 40 min	Probe sonication; filtration	37 ± 04	−27 ± 01	0.47	–OH	Negative
**CNP-PEG 1 K**	Pre-passivation	1:10	water	270 °C; 40 min	Probe sonication; filtration	08 ± 01	−22 ± 04	0.53	–OH	Negative
**CNP-PEG 4.6 K**	Pre-passivation	1:10	water	270 °C; 40 min	Probe sonication; filtration	139 ± 07	−27 ± 03	0.22	–OH	Negative
**CNP-PEG 10k**	Pre-passivation	1:10	water	270 °C; 40 min	Probe sonication; filtration	28 ± 03	−22 ± 02	0.23	–OH	Negative
**CNP-PEG 20k**	Pre-passivation	1:10	water	270 °C; 40 min	Probe sonication; filtration	02 ± 01	−24 ± 03	0.48	–OH	Negative
**CNP-Bis-MPA**	Post-passivation	1:10	water	270 °C; 30 min then RT	Probe sonication; filtration; incubation	190 ± 80	−25 ± 01	0.66	–OH	Negative
**CNP-PAMAM−**	Post-passivation	1:10	water	270 °C; 30 min then RT	Probe sonication; filtration; incubation	200 ± 50	−35 ± 01	0.55	–COOH	Negative
**CNP-PAMAM+**	Post-passivation	1:10	water	270 °C; 30 min then RT	Probe sonication; filtration; incubation	243 ± 19	26 ± 05	0.56	–NH2	positive
**CNP-PolyLys**	Post-passivation	1:10	water	270 °C; 30 min then RT	Probe sonication; filtration; incubation	219 ± 22	16 ± 06	0.64	–NH2	Positive
**CNP-PEI**	Post-passivation	1:10	water	270 °C; 30 min then RT	Probe sonication; filtration; incubation	41 ± 21	09 ± 01	0.60	–NH2	Positive

Percent distribution of hydrodynamic diameters (number averaged, nm, 0.2 *μ*M nanopure water) and polydispersity indexes were obtained from dynamic light scattering (DLS).

^*^Preparation temperature and time: Pre-passivated particles were prepared at 270 °C followed by post synthetic processing; whereas post-passivated particles were incubated with the passivating agent at ambient temperature.

^**^Post synthesis processing: 20-minute probe sonication; Amp 1, On: 2 Sec, Off: 1 Sec, followed by filtration through 0.2 *μ* cellulosic membrane.

**Table 2:** Numerical results of CH50, IC 50 and P values.

Samples	Reference/control 1	Reference/control 2	PEI 25 K	CNP-PEI	CNP-PAMAM+	CNP-PolyLys	CNP-PEG400	CNP-PEG1 K	CNP-PEG4.6 K	CNP-PEG10 K	CNP-PEG20 K	CNP-Bis-MPA	CNP-PAMAM−
CH50	9 ± 1	2 ± 1	2 ± 1	5 ± 1	5 ± 1	6 ± 1	8 ± 1	8 ± 1	8 ± 1	8 ± 1	8 ± 1	8 ± 1	8 ± 1
P Value	Standard	<0.001; ***	<0.001; ***	<0.005; **	<0.005; **	<0.005; **	>0.05; NS	>0.05; NS	>0.05; NS	>0.05; NS	>0.05; NS	>0.05; NS	>0.05; NS
IC_50_ μg/ml	—	—	<<30	400 ± 20	100 ± 20	300 ± 30	1500 ± 100	2000 ± 200	2000 ± 200	1400 ± 100	1200 ± 50	1000 ± 50	1000 ± 50

The significance levels were presented by P values. *P<0.05, **P<0.01, ***P<0.001, ****P<0.0001. NS stands for not statistically significant and IC50 values for CNP-PEG400, CNP-PEG1K, CNP-PEG4.6K, CNP-PEG10K and CNP-PEG20K are not represented in the plot.

## References

[b1] Kamaly N., Xiao Z., Valencia P. M., Radovic-Moreno A. F. & Farokhzad O. C. Targeted polymeric therapeutic nanoparticles: Design, development and clinical translation. Chem. Soc. Rev. 41, 2971–3010 (2012). 22388185 10.1039/c2cs15344k PMC3684255

[b2] Li K. & Liu B. Polymer-encapsulated organic nanoparticles for fluorescence and photoacoustic imaging. Chem. Soc. Rev. 43, 6570–6597 (2014). 24792930 10.1039/c4cs00014e

[b3] Jain P., Huang X., El-Sayed I. & El-Sayed M. Noble metals on the nanoscale: Optical and photothermal properties and some applications in imaging, sensing, biology, and medicine. Acc. Chem. Res. 41, 1578–1586 (2008). 18447366 10.1021/ar7002804

[b4] Huang X., El-Sayed I., Qian W. & El-Sayed M. Cancer cell imaging and photothermal therapy in the near-infrared region by using gold nanorods. J. Am. Chem. Soc. 128, 2115–2120 (2006). 16464114 10.1021/ja057254a

[b5] Moghimi S., Hunter A. & Murray J. Nanomedicine: Current status and future prospects. FASEB J. 19, 311–330 (2005). 15746175 10.1096/fj.04-2747rev

[b6] Zhang L. *et al.* Nanoparticles in medicine: Therapeutic applications and developments. Clin. Pharmacol. Ther. 83, 761–769 (2008). 17957183 10.1038/sj.clpt.6100400

[b7] Yah C., Simate G. & Iyuke S. Nanoparticles toxicity and their routes of exposures. Pak. J. Pharm. Sci. 25, 477 (2012). 22459480

[b8] Qureshi S. & Medzhitov R. Toll-like receptors and their role in experimental models of microbial infection. Genes Immun. 4, 87–94 (2003). 12618855 10.1038/sj.gene.6363937

[b9] Kraft J., Freeling J., Wang Z. & Ho R. Emerging research and clinical development trends of liposome and lipid nanoparticle drug delivery systems. J. Pharm. Sci. 103, 29–52 (2014). 24338748 10.1002/jps.23773 PMC4074410

[b10] Dash B. *et al.* The influence of size and charge of chitosan/polyglutamic acid hollow spheres on cellular internalization, viability and blood compatibility. Biomaterials 31, 8188–8197 (2010). 20701967 10.1016/j.biomaterials.2010.07.067

[b11] Hamad I. *et al.* Distinct polymer architecture mediates switching of complement activation pathways at the nanosphereserum interface: Implications for stealth nanoparticle engineering. ACS Nano 4, 6629–6638 (2010). 21028845 10.1021/nn101990a

[b12] Salvador-Morales C., Zhang L., Langer R. & Farokhzad O. Immunocompatibility properties of lipid-polymer hybrid nanoparticles with heterogeneous surface functional groups. Biomaterials 30, 2231–2240 (2009). 19167749 10.1016/j.biomaterials.2009.01.005 PMC2699891

[b13] Rolland A., Collet B., Le Verge R. & Toujas L. Blood clearance and organ distribution of intravenously administered polymethacrylic nanoparticles in mice. J. Pharm. Sci. 78, 481–484 (1989). 2760823 10.1002/jps.2600780613

[b14] Dobrovolskaia M. & McNeil S. Immunological properties of engineered nanomaterials. Nat. Nanotechnol. 2, 469–478 (2007). 18654343 10.1038/nnano.2007.223

[b15] Andersen A. *et al.* Single-walled carbon nanotube surface control of complement recognition and activation. ACS Nano 7, 1108–1119 (2013). 23301860 10.1021/nn3055175

[b16] Hooshmand S. *et al.* Effects of agave nectar versus sucrose on weight gain, adiposity, blood glucose, insulin, and lipid responses in mice. J. Med. Food 17, 1017–1021 (2014). 25011004 10.1089/jmf.2013.0162

[b17] Bhagat K. B. D., Patil P. & Paknikar K. Hydrothermal synthesis and characterization of carbon nanospheres: a mechanistic insight. RSC Adv. 5, 59491–59494 (2015).

[b18] Wu L. *et al.* A green synthesis of carbon nanoparticle from honey for real-time photoacoustic imaging. Nano Res. 6, 312–325 (2013). 23824757 10.1007/s12274-013-0308-8 PMC3696503

[b19] Sharma M. Understanding the mechanism of toxicity of carbon nanoparticles in humans in the new millennium: A systemic review. Indian J. Occup. Environ. Med., 14, 3–5 (2010). 20808660 10.4103/0019-5278.64607 PMC2923423

[b20] Godbey K. K., Wu W. T. & Mikos A. G. Poly(ethyleneimine)-mediated gene delivery affects endothelial cell function and viability. Biomaterials 22, 471–480 (2001). 11214758 10.1016/s0142-9612(00)00203-9

[b21] Xue S., Liu H. Y. & Wong H. L. Nanotoxicity: a key obstacle to clinical translation of sirna-based nanomedicine. Nanomedicine (Lond). 9, 295–312 (2014). 24552562 10.2217/nnm.13.204 PMC4095781

[b22] Fischer D., Bieber Y., Li T. & Kissel T. A novel non-viral vector for dna delivery based on low molecular weight, branched polyethylenimine: effect of molecular weight on transfection efficiency and cytotoxicity. Pharm. Res. 16, 1273–1279 (1999). 10468031 10.1023/a:1014861900478

[b23] Jäger M., Schubert S. F. D., Ochrimenko S. & Schubert U. S. Branched and linear poly(ethylene imine)-based conjugates: synthetic modification, characterization, and application. Chem. Soc. Rev. 41, 4755–4767 (2012). 22648524 10.1039/c2cs35146c

[b24] Merkel O. M. *et al.* *In vitro* and *in vivo* complement activation and related anaphylactic effects associated with polyethylenimine and polyethylenimine-graft-poly(ethylene glycol) block copolymers. Biomaterials 32, 4936–4942 (2011). 21459440 10.1016/j.biomaterials.2011.03.035

[b25] Pasut G. & Veronese F. M. State of the art in pegylation: the great versatility achieved after forty years of research. J. Control. Release 161, 461–472 (2012). 22094104 10.1016/j.jconrel.2011.10.037

[b26] Moghimi S. M. *et al.* Complement activation cascade triggered by peg-pl engineered nanomedicines and carbon nanotubes: the challenges ahead. J. Control. Release 146, 175–181 (2010). 20388529 10.1016/j.jconrel.2010.04.003

[b27] Plank C., Mechtler K., Szoka J. F. C. & Wagner E. Activation of the complement system by synthetic dna complexes: A potential barrier for intravenous gene delivery. Hum. Gene Ther. 7, 1437–1446 (1996). 8844203 10.1089/hum.1996.7.12-1437

[b28] Mukherjee S. P., M. D. & Byrne H. J. *In vitro* mammalian cytotoxicological study of pamam dendrimers-towards quantitative structure activity relationships. Toxicol. in Vitro 24, 169–177 (2010). 19778601 10.1016/j.tiv.2009.09.014

[b29] Naha M. L. F. M., Davoren P. C. & Byrne H. J. Reactive oxygen species (ros) induced cytokine production and cytotoxicity of pamam dendrimers in j774a.1 cells. Toxicol. Appl. Pharmacol. 246, 91–99 (2010). 20420846 10.1016/j.taap.2010.04.014

[b30] McNerny D. Q., Leroueil P. R. & Baker J. R. Understanding specific and nonspecific toxicities: a requirement for the development of dendrimer-based pharmaceuticals. Nanomed. Nanobiotechnol. 2, 249–259 (2010). 10.1002/wnan.79 PMC2905802 20166124

[b31] Fröhlich E. The role of surface charge in cellular uptake and cytotoxicity of medical nanoparticles. Int. J. Nanomedicine 7, 5577–5591 (2012). 23144561 10.2147/IJN.S36111 PMC3493258

[b32] Zeng X., Morgenstern R. & Nyström A. M. Nanoparticle-directed sub-cellular localization of doxorubicin and the sensitization breast cancer cells by circumventing gst-mediated drug resistance. Biomaterials 35, 1227–1239 (2014). 24210875 10.1016/j.biomaterials.2013.10.042

[b33] Parkin J. & Cohen B. An overview of the immune system. Lancet 357, 1777–1789 (2001). 11403834 10.1016/S0140-6736(00)04904-7

[b34] Walport M. Advances in immunology: Complement (first of two parts). N. Engl. J. Med. 344, 1058–1066 (2001). 11287977 10.1056/NEJM200104053441406

[b35] Pham C. *et al.* Variable antibody-dependent activation of complement by functionalized phospholipid nanoparticle surfaces. J. Biol. Chem. 286, 123–130 (2011). 21047788 10.1074/jbc.M110.180760 PMC3012966

[b36] Ricklin D., Hajishengallis G., Yang K. & Lambris J. Complement: A key system for immune surveillance and homeostasis. Nat. Immunol. 11, 785–797 (2010). 20720586 10.1038/ni.1923 PMC2924908

[b37] Moghimi S. *et al.* Material properties in complement activation. Adv. Drug Delivery Rev. 63, 1000–1007 (2011). 10.1016/j.addr.2011.06.002 21689701

[b38] Moghimi S. & Szebeni J. Stealth liposomes and long circulating nanoparticles: Critical issues in pharmacokinetics, opsonization and protein-binding properties. Prog. Lipid Res. 42, 463–478 (2003). 14559067 10.1016/s0163-7827(03)00033-x

[b39] Chonn A., Cullis P. & Devine D. The role of surface charge in the activation of the classical and alternative pathways of complement by liposomes. J. Immunol. 146, 4234–4241 (1991). 2040798

[b40] Merkel O. M. *et al.* *In vitro* and *in vivo* complement activation and related anaphylactic effects associated with polyethylenimine and polyethylenimine-graft-poly(ethylene glycol) block copolymers. Biomaterials 32, 4936–4942 (2011). 21459440 10.1016/j.biomaterials.2011.03.035

[b41] Ogris M., Brunner S., Schüller S., Kircheis R. & Wagner E. PEGylated DNA/transferrin-PEI complexes: Reduced interaction with blood components, extended circulation in blood and potential for systemic gene delivery. Gene Ther. 6, 595–605 (1999). 10476219 10.1038/sj.gt.3300900

